# [^18^F]Flotaza for Aβ Plaque Diagnostic Imaging: Evaluation in Postmortem Human Alzheimer’s Disease Brain Hippocampus and PET/CT Imaging in 5xFAD Transgenic Mice

**DOI:** 10.3390/ijms25147890

**Published:** 2024-07-18

**Authors:** Yasmin K. Sandhu, Harman S. Bath, Jasmine Shergill, Christopher Liang, Amina U. Syed, Allyson Ngo, Fariha Karim, Geidy E. Serrano, Thomas G. Beach, Jogeshwar Mukherjee

**Affiliations:** 1 Preclinical Imaging, Department of Radiological Sciences, University of California-Irvine, Irvine, CA 92697, USA; sandhuy@uci.edu (Y.K.S.); bathhs@uci.edu (H.S.B.); jshergi1@uci.edu (J.S.); liangc@uci.edu (C.L.); ausyed@uci.edu (A.U.S.); allyson1@uci.edu (A.N.); fkarim1@uci.edu (F.K.); 2 Banner Sun Health Research Institute, Sun City, AZ 85351, USA; geidy.serrano@bannerhealth.com (G.E.S.); thomas.beach@bannerhealth.com (T.G.B.)

**Keywords:** [^18^F]flotaza, human Aβ plaques, hippocampus, Alzheimer’s disease, PET imaging, 5xFAD transgenic mice

## Abstract

The diagnostic value of imaging Aβ plaques in Alzheimer’s disease (AD) has accelerated the development of fluorine-18 labeled radiotracers with a longer half-life for easier translation to clinical use. We have developed [^18^F]flotaza, which shows high binding to Aβ plaques in postmortem human AD brain slices with low white matter binding. We report the binding of [^18^F]flotaza in postmortem AD hippocampus compared to cognitively normal (CN) brains and the evaluation of [^18^F]flotaza in transgenic 5xFAD mice expressing Aβ plaques. [^18^F]Flotaza binding was assessed in well-characterized human postmortem brain tissue sections consisting of HP CA1-subiculum (HP CA1-SUB) regions in AD (n = 28; 13 male and 15 female) and CN subjects (n = 32; 16 male and 16 female). Adjacent slices were immunostained with anti-Aβ and analyzed using QuPath. In vitro and in vivo [^18^F]flotaza PET/CT studies were carried out in 5xFAD mice. Post-mortem human brain slices from all AD subjects were positively IHC stained with anti-Aβ. High [^18^F]flotaza binding was measured in the HP CA1-SUB grey matter (GM) regions compared to white matter (WM) of AD subjects with GM/WM > 100 in some subjects. The majority of CN subjects had no decipherable binding. Male AD exhibited greater WM than AD females (AD WM♂/WM♀ > 5; *p* < 0.001) but no difference amongst CN WM. In vitro studies in 5xFAD mice brain slices exhibited high binding [^18^F]flotaza ratios (>50 versus cerebellum) in the cortex, HP, and thalamus. In vivo, PET [^18^F]flotaza exhibited binding to Aβ plaques in 5xFAD mice with SUVR~1.4. [^18^F]Flotaza is a new Aβ plaque PET imaging agent that exhibited high binding to Aβ plaques in postmortem human AD. Along with the promising results in 5xFAD mice, the translation of [^18^F]flotaza to human PET studies may be worthwhile.

## 1. Introduction

The amyloid cascade hypothesis in Alzheimer’s disease (AD) continues to play a significant role in the potential management of the illness [1,2,3,4,5]. Clinical imaging of Aβ plaques in AD using positron emission tomography (PET) has accelerated with the development of longer-lasting diagnostic half-life radiotracers [6,7,8]. Furthermore, the demonstration of the therapeutic value of Aβ plaque load reduction using Aβ plaque imaging is being actively pursued [9,10]. Because of this significant diagnostic and therapeutic value, the development of improved fluorine-18 labeled Aβ plaque imaging agents has continued in attempts to enhance standard uptake value ratios (SUVRs, a measure of Aβ plaque load in the grey matter versus the white matter) in the cortex of AD patients. A significantly higher SUVR in the AD cortex may be valuable in increasing the sensitivity to detect change and, thus, make more accurate assessments of diagnostic and therapeutic value [6,11].

A good understanding of the progression of Aβ plaques in the human AD brain has now been achieved by using several different PET radiotracers. The formation of Aβ plaques occurs early within the temporal lobe, including the hippocampus and entorhinal cortex [12]. Neocortical regions, including the cingulate gyrus, are subsequently significantly affected by the accumulation of Aβ plaques. The amount of Aβ plaque load in various brain regions is very high based on postmortem human AD brain studies. However, because of the nonspecific white matter (WM) binding of many of the PET radiotracers, the value of SUVRs reported rarely exceeds two in advanced AD. Currently, several PET radiotracers ([^18^F]florbetaben, [^18^F]florbetapir, and [^18^F]flutemetamol) are being used in clinical research, and efforts are still underway in order to further improve in vivo imaging properties [6].

**Figure 1:**
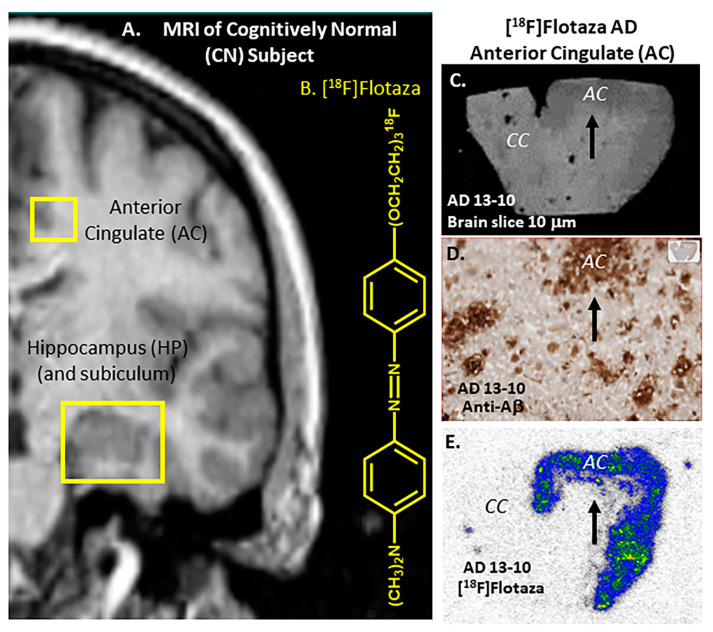
[^18^F]Flotaza for imaging human Aβ plaques in AD subjects: (**A**) MRI coronal brain slice of CN subject [16] showing brain regions of anterior cingulate (AC) and hippocampus (HP); (**B**) Chemical structure of [^18^F]flotaza; (**C**) Postmortem brain slice (10 μm) of AC (arrow), including the corpus callosum (CC) from AD subject (AD 13-10) showing gray matter (GM) and white matter (WM); (**D**) Anti-Aβ immunostained brain slice of AD 13-10 showing presence of Aβ plaques in AC (arrow; inset shows whole slice); (**E**) [^18^F]Flotaza binding to Aβ plaques in the AC GM regions (arrow) of AD 13-10 subject [17].

To further identify additional PET radiotracers that may be able to provide higher SUVRs, we have continued the search for more optimal candidate PET radiotracers. We have explored PET radiotracers for imaging Aβ plaques with the goal of reducing WM binding [13,14,15]. In this effort, we have successfully developed and evaluated the binding of [^18^F]flotaza in postmortem human AD [14]. [^18^F]Flotaza selectively binds to human Aβ plaques with high affinity (Ki = 1.68 nM) and has a very weak affinity (Ki > 10 mM) for Tau protein (present in neurofibrillary tangles, NFT), which has previously been identified using [^125^I]IPPI. Figure 1A shows a coronal MRI brain slice of a control subject depicting brain regions studied with [^18^F]flotaza (Figure 1B) [16]. In our previous human AD postmortem studies, anterior cingulate regions, including the corpus callosum of the brain, were examined using [^18^F]flotaza (Figure 1C,E). Quantitative QuPath analysis of anti-Aβ immunostained slices (Figure 1D) with [^18^F]flotaza autoradiographs provided good measures of Aβ plaque load in all the subjects with very high ratios between anterior cingulate grey matter (GM) regions and the corpus callosum WM regions [17]. The control subject anterior cingulate exhibited no binding of [^18^F]flotaza. The unique diaza structural feature of [^18^F]flotaza resulted in high in vitro post-mortem brain ratios. This is indicative of the good prospects of [^18^F]flotaza serving as a PET imaging agent for Aβ plaques.

In order to further ascertain the properties of [^18^F]flotaza in a different brain region, we have now evaluated the binding of [^18^F]flotaza in male and female human postmortem hippocampus (CA1 plus subiculum) in cognitively normal control (CN) and AD subjects, which has been shown to have higher levels of Aβ plaques compared to other regions of the hippocampus [18]. [^18^F]Flotaza binding was quantitatively compared with anti-Aβ immunostained sections. [^18^F]Flotaza was also evaluated in transgenic 5xFAD mice brains, which express Aβ plaques and are useful models of human AD [19,20,21]. In vitro brain studies, PET/CT in vivo imaging, and ex vivo autoradiographic analyses of brain slices were carried out in the 5xFAD mice in order to evaluate the suitability of [^18^F]flotaza for in vivo PET imaging of Aβ plaques.

## 2. Results

### 2.1. Female AD Human Postmortem Subjects

**Figure 2:**
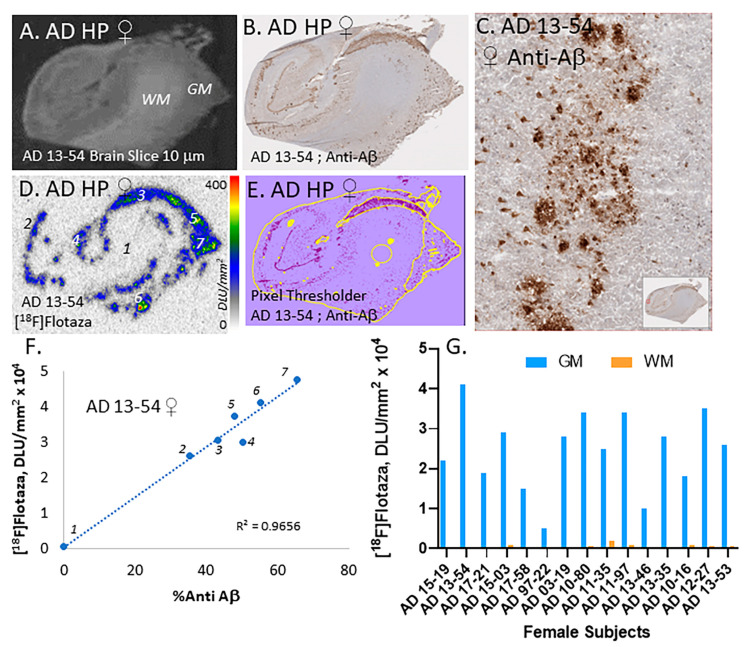
[^18^F]Flotaza in HP of female AD subjects: (**A**) AD brain slice of female subject 13-54 showing GM and WM in human HP; (**B**) Anti-Aβ immunostained adjacent 13-54 section showing presence of Aβ plaques; (**C**) Aβ plaques seen at 100 μm magnification; (**D**) [^18^F]Flotaza binding in the GM (#2–7) and WM (#1) regions in adjacent slices; (**E**) Anti-Aβ plaque pixel classifier image (yellow border) of immunostained 13-54 brain slice; (**F**) Correlation (R^2^ = 0.97) of [^18^F]flotaza binding and Aβ plaque load in different regions (#1–7) of the brain slice (seen in (**D**,**E**)); (**G**) High [^18^F]flotaza binding in GM of 15 female AD subjects with very little WM binding.

Figure 2A shows the scan of the CA1 subiculum of one female AD subject, showing the GM and WM regions. Extensive amounts of neurotic, cored, and diffuse Aβ plaques (Figure 2B,C) were immunostained and consistent with previous reports of high levels of immunostained Aβ plaques in this brain region [19,22]. The brain slice in Figure 2A was labeled by [^18^F]flotaza and shows high levels of binding in the GM, while the WM regions were near background levels (Figure 2D). Analysis by QuPath of the immunostained slice in Figure 2B provided the pixel threshold image shown in Figure 2E. This was used to provide a quantitative assessment of Aβ positivity, which was plotted using quantitative measures of [^18^F]flotaza binding (Figure 2F; [17]). The regions of WM and GM (regions 1 through 7 highlighted in Figure 2D) were plotted, showing a high correlation between [^18^F]flotaza binding and Aβ positivity within the brain slice.

As seen in Figure 2G, all female AD subjects (n = 15) exhibited high [^18^F]flotaza binding in the GM regions and very low WM binding in the hippocampus. When compared to the other subjects, two subjects (AD 97-22 and AD 13-46) had lower levels of GM binding. Since all subjects were expected to have high Aβ plaque load (Table 1), these deviations may be due to heterogeneity within the specimens. Figure 2B,D,F suggest that the subiculum also has high levels of [^18^F]flotaza binding and is consistent with previous Aβ immunostain findings of the human subiculum [23]. It may also be noted that some of the variability in the [^18^F]flotaza binding may be due to variations in the tissue sections.

### 2.2. Male AD Human Postmortem Subjects

**Figure 3:**
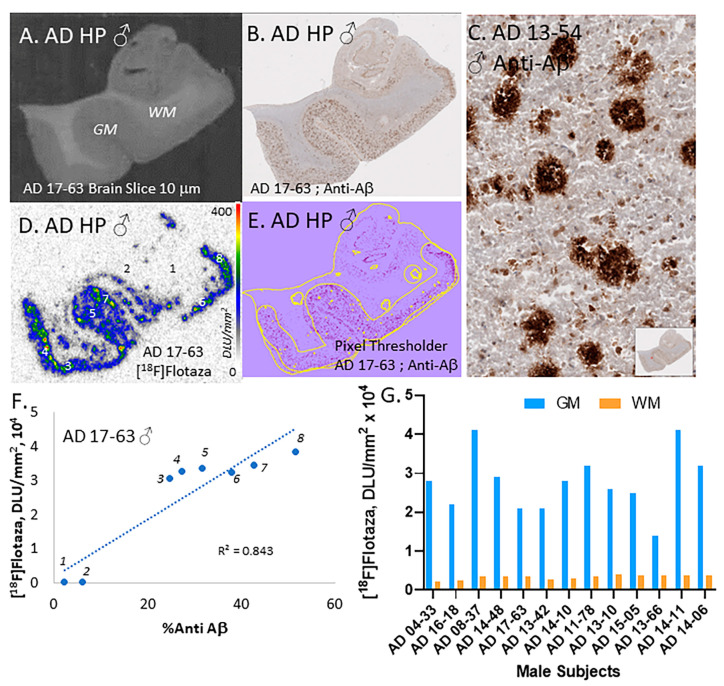
[^18^F]Flotaza in HP of male AD subjects: (**A**) AD brain slice of male subject 17-63 showing GM and white matter WM in human HP; (**B**) Anti-Aβ immunostained adjacent 17-63 section showing presence of Aβ plaques; (**C**) Aβ plaques seen at 100 μm magnification (inset shows brain slice); (**D**) [^18^F]Flotaza binding in the GM (#3–8) and WM (#1,2) regions in adjacent slices; (**E**) Anti-Aβ plaque pixel classifier image (yellow border) of immunostained 17-63 brain slice; (**F**) Correlation (R^2^ = 0.84) of [^18^F]flotaza binding and Aβ plaque load in different regions (#1–8) of the brain slice (seen in (**D**,**E**)); (**G**) High [^18^F]flotaza binding in GM of 13 male AD subjects with lower WM binding.

Male AD subjects also revealed extensive amounts of Aβ plaques. One subject shown in Figure 3B,C had an abundant amount of neurotic, cored, and diffuse Aβ plaques (Figure 3B,C). [^18^F]Flotaza was able to consistently bind to these Aβ plaque sites (Figure 3D). Through QuPath analysis, a generated pixel threshold image shown in Figure 3E was able to provide a quantitative assessment of Aβ positivity, which was plotted with quantitative measures of [^18^F]flotaza binding (Figure 3F). Both WM and GM regions (regions 1 through 8 highlighted in Figure 3D) were plotted with a high correlation between [^18^F]flotaza binding and Aβ positivity. All male AD subjects (n = 13) exhibited high [^18^F]flotaza binding in the GM regions, as seen in Figure 3G. Male AD subjects exhibited greater binding of [^18^F]flotaza in the WM regions compared to the female subjects. This higher WM binding (and the lower WM binding in female subjects) appeared to be consistent in all the subjects (Figure 2G and Figure 3G). Similar to the female subjects, some of the variability in the [^18^F]flotaza binding may be due to variations in the tissue sections.

### 2.3. Comparing Male and Female AD Human Postmortem Subjects

**Figure 4:**
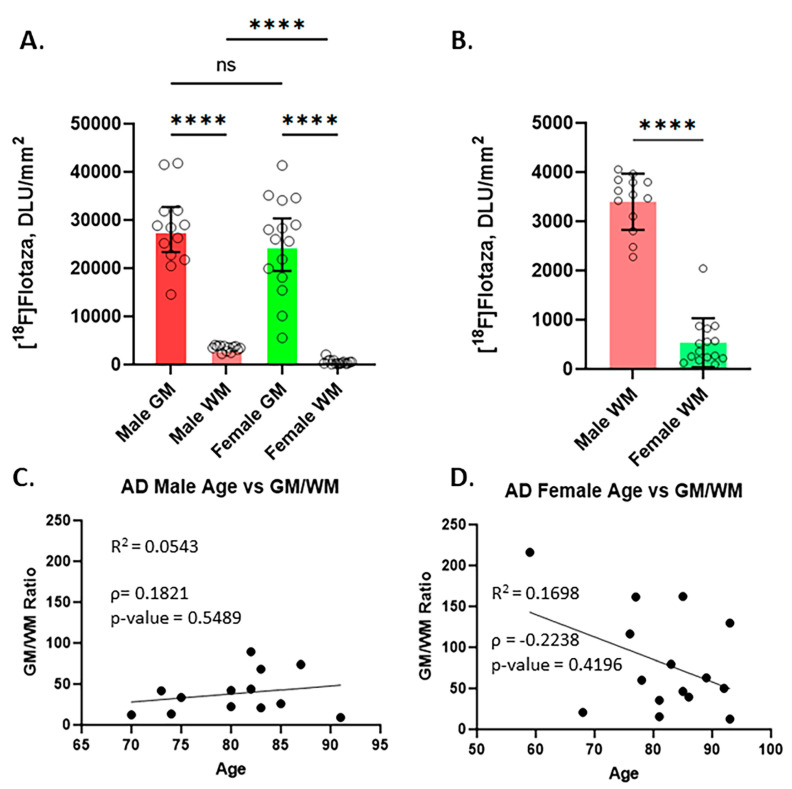
Comparison of [^18^F]flotaza in HP of male and female AD subjects: (**A**) Plot showing the average of GM and WM of 13 male subjects and the average of GM and WM of 15 female subjects. The large difference between [^18^F]flotaza binding in GM and WM in both male and female subjects was highly significant (**** *p* < 0.001). Differences in male GM and female GM were not significant; (**B**) [^18^F]Flotaza binding to WM in males and females was significantly different (**** *p* < 0.001), with males exhibiting higher binding compared to females WM; (**C**) Male GM/WM ratio of [^18^F]flotaza versus age (R^2^ = 0.0543; Spearman’s correlation ρ = 0.1821, *p*-value = 0.5489); (**D**) Female GM/WM ratio of [^18^F]flotaza versus age (R^2^ = 0.1698; Spearman’s correlation ρ = −0.2238, *p*-value = 0.4196).

The extent of [^18^F]flotaza binding in male and female subjects GM was similar, and the difference was not statistically significant, as seen in Figure 4A. A lack of male–female differences have been previously reported in postmortem hippocampal findings using immunostaining methods [23]. As expected, the difference between GM and WM in all males and all females was highly significant (*p* < 0.001, Figure 4A). Interestingly, the WM difference between males and females was highly significant (*p* < 0.001), with females showing significantly lower WM binding (Figure 4B). The greater WM binding in males resulted in lower GM/WM ratios (Figure 4C) compared to the female GM/WM ratios, which were higher because of the lower WM binding (Figure 4D). The GM/WM ratio in male AD subjects ranged from 9.2 to 89.3, whereas in the female AD subjects, it ranged from 12.5 to 216. There was a poor correlation of age with the [^18^F]flotaza GM/WM ratios both for males (R^2^ = 0.0543; Spearman’s correlation ρ = 0.1821, *p*-value = 0.5489; Figure 4C) and females (R^2^ = 0.1698; Spearman’s correlation ρ = −0.2238, *p*-value = 0.4196; Figure 4D).

### 2.4. Cognitively Normal (CN) Human Postmortem Subjects

**Figure 5:**
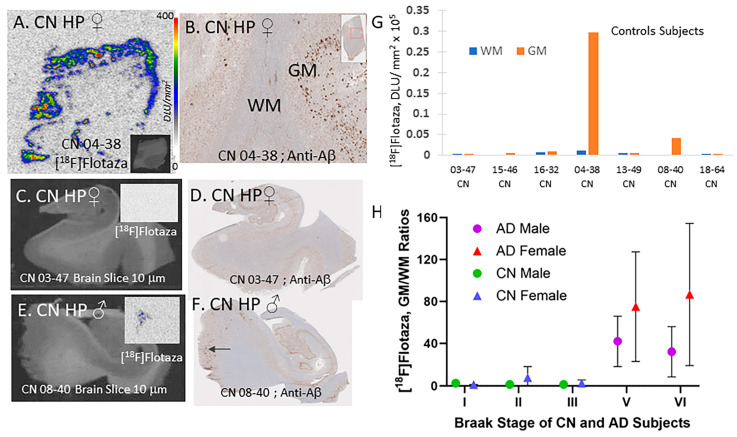
[^18^F]Flotaza in HP of CN subjects: (**A**) [^18^F]Flotaza binding in brain slice of female CN 04-38 subject showing greater binding in GM compared to WM (inset shows scan of brain slice); (**B**) Anti-Aβ immunostained adjacent section of CN 04-38 showing presence of Aβ plaques (inset shows whole slice); (**C**) Hippocampus brain slice CN female subject, CN 03-47 (inset shows no [^18^F]flotaza binding in the GM); (**D**) Anti-Aβ immunostained adjacent section of CN 03-47 showing absence of Aβ plaques; (**E**) Hippocampus brain slice CN male subject, CN 08-40 (inset shows small amount of [^18^F]flotaza binding in the GM); (**F**) Anti-Aβ immunostained adjacent section of CN 08-40 showing mostly absence of Aβ plaques, except regions shown by arrow; (**G**) Out of 32 CN subjects, 7 subjects exhibit some [^18^F]flotaza binding, with two subjects (04-38 and 08-40) showing significant levels of GM binding; (**H**) Plot of [^18^F]flotaza GM/WM binding ratio in the HP of all CN and AD with respect to Braak Staging of the subjects. Braak stages V and VI showed high ratios, with females exhibiting greater ratios compared to males.

Of the 32 CN subjects used in the study, only one subject had significant [^18^F]flotaza binding (Figure 5A,B). About five subjects showed some decipherable binding above the background without any GM binding preference (Figure 5C,D), while other subjects had some decipherable GM binding higher than WM binding (Figure 5E,F). All other control subjects had no binding whatsoever, suggesting the high specificity of [^18^F]flotaza binding to Aβ plaques. The absence of Aβ plaques in the brain slices of these remaining CN subjects was confirmed by the absence of any anti-Aβ immunostaining.

All the subjects (CN and AD) used in this study were categorized in Braak stages I, II, III, V, and VI (none of the subjects were found to be in the Braak IV stage). Figure 5H shows a plot of [^18^F]flotaza GM/WM binding ratio in all the subjects with respect to Braak staging. Females had the highest ratios, with Braak stage VI having the maximum average (average ratios ~80). Similarly, males had high ratios in Braak stages V and VI, but the average ratio was about half of those found for females. As indicated before, the lower male ratio of GM/WM is driven by the higher WM binding seen in males compared to the female AD subjects. Braak stages I–III did not have significant [^18^F]flotaza binding.

### 2.5. Transgenic 5xFAD Mice In Vitro

**Figure 6:**
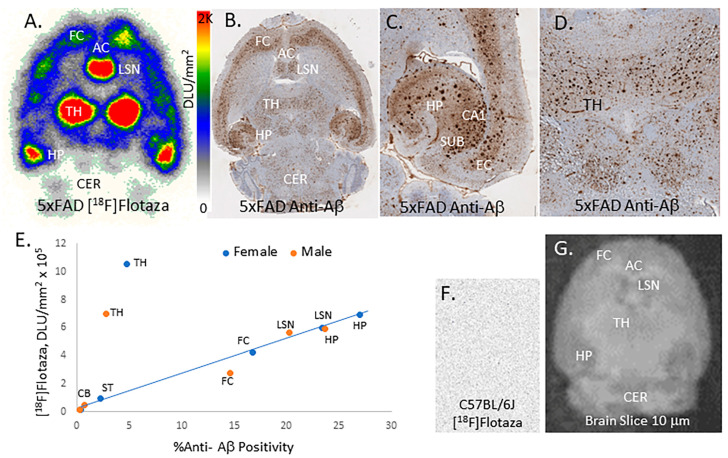
In vitro [^18^F]flotaza in transgenic 5xFAD mice: (**A**) [^18^F]Flotaza binding in 10 μm-thick horizontal brain slice of female transgenic 5xFAD mouse (FC: frontal cortex; AC: anterior cingulate; LSN: lateral septal nucleus; TH: thalamus; HP: hippocampus; CER: cerebellum); (**B**) Anti-Aβ immunostained adjacent section of 5xFAD mouse showing the presence of Aβ plaques; (**C**) Close-up (100 μm) of HP regions, CA1, subiculum (SUB) and entorhinal cortex (EC) showing Aβ plaques; (**D**) Close-up (100 μm) of TH showing more diffuse Aβ plaques; (**E**) Plot of [^18^F]flotaza in vitro binding in different brain regions versus the %anti-Aβ positivity in the 5xFAD brain slices. (**F**) Absence of [^18^F]flotaza binding in 10 μm-thick horizontal brain slice of C57BL/6 WT mouse; (**G**) Horizontal brain slice, 10 μm-thick of C57BL/6 WT mouse.

Transgenic 5xFAD mice and wild-type C57BL/6J mice brain slices were used to evaluate the total binding of [^18^F]flotaza. The imaging protocol for the autoradiographic study of mice was similar to that of postmortem human brains. These brain slices had the frontal cortex, lateral septal nuclei, thalamus, hippocampus, and cerebellum (Figure 6). Extensive binding of [^18^F]flotaza was observed in cortical regions, including the frontal cortex and anterior cingulate, lateral septal nuclei, thalamus, and hippocampus, with very little binding in the cerebellum (Figure 6A). The ranking order of binding was: thalamus > lateral septal nuclei = hippocampus > frontal cortex > striatum > cerebellum. Both male and female mice had a similar order of [^18^F]flotaza binding (Figure 6E).

The binding of [^18^F]flotaza in the various brain regions strongly correlated with anti-Aβ immunostains for Aβ plaques, hence confirming the binding of [^18^F]flotaza to the regions that contained high Aβ deposition (Figure 6B). Closer inspection of the hippocampal region (Figure 6C) revealed high Aβ positivity, with a significant amount of [^18^F]flotaza occurring in the CA1 and subiculum regions. The thalamus Aβ positivity (Figure 6D) was more diffuse in nature and not as high as the hippocampus, but the binding of [^18^F]flotaza in the thalamus was very high. There was a good correlation between Aβ positivity measured in the adjacent immunostained sections and [^18^F]flotaza binding in the different brain regions, except for the thalamus, which appears as an outlier with high levels of [^18^F]flotaza binding (Figure 6E). In the wild-type C57BL/6J mice brain slices lacking the Aβ plaques, there was no [^18^F]flotaza binding (Figure 6F,G).

The ratio of [^18^F]flotaza binding in different brain regions versus the cerebellum as a reference region was high. In descending order, the ratios for females and males were: thalamus (TH/CB = 65♀; 41♂), hippocampus (HP/CB = 43♀; 35♂), lateral septal nuclei (LSN/CB = 37♀; 33♂), frontal cortex (FC/CB = 26♀; 16♂), and striatum (ST/CB = 6♀; 3♂). In this preliminary in vitro study, female mice showed higher ratios in all brain regions compared to the males. The order of [^18^F]flotaza binding ratios in the different brain regions was the same for females and males.

### 2.6. PET/CT Imaging of Transgenic 5xFAD Mice

**Figure 7:**
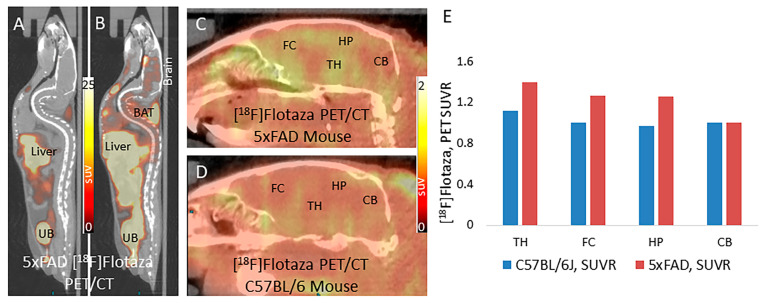
In vivo [^18^F]flotaza in transgenic 5xFAD mice: (**A**) In vivo PET/CT whole body sagittal image of 5xFAD mouse (12-month-old) showing binding of [^18^F]flotaza after retroorbital administration of 2.63 MBq of [^18^F]flotaza (UB: urinary baldder); (**B**) Lower threshold image showing PET binding of [^18^F]flotaza in the brain (UB: urinary bladder; BAT: brown adipose tissue); (**C**) Brain in vivo PET/CT sagittal image of the 5xFAD mouse showing binding of [^18^F]flotaza in Aβ-rich regions (FC: frontal cortex; TH: thalamus; HP: hippocampus) and reference cerebellum (CB); (**D**) In vivo PET/CT brain sagittal image of C57BL/6 mouse (12-month-old) showing lack of binding of [^18^F]flotaza within the brain after retroorbital administration of 3.07 MBq; (**E**) Plot shows standard uptake value ratio (SUVR) with respect to cerebellum of in vivo PET/CT [^18^F]flotaza in 5xFAD mice and the WT C57BL/6 mouse. Higher ratios in TH, FC, and HP (1.2 to 1.45) were seen in the 5xFAD mice compared to the C57BL/6 mice.

Mice (5xFAD and C57BL/6J) were injected retro-orbitally under anesthesia with [^18^F]flotaza and tolerated the procedure well. Intraperitoneal administration of [^18^F]flotaza was not suitable because of its lipophilicity, causing insufficient absorption in the bloodstream. Retro-orbital administration allowed sufficient distribution, although a significant amount of [^18^F]flotaza radioactivity remained in the eye orbit. After an uptake period of 90 min post-injection, a whole-body PET/CT scan was acquired on both groups of mice. Figure 7A shows a sagittal whole-body distribution of [^18^F]flotaza in the 5xFAD mouse post retro-orbital administration. Major organs that accumulated the radioactivity included the liver, urinary bladder, kidneys, and gastrointestinal tract. Inset shows an image with a lower PET intensity to demarcate the liver and urinary bladder. This distribution of [^18^F]flotaza is similar to reports for other pegylated derivatives [21,26], where clearance of the radiotracer is seen via the kidney and the gastrointestinal tract to the urinary bladder. The uptake of [^18^F]flotaza in the brain is lower compared to the rest of the body. An intravenous injection of [^18^F]flotaza may help provide greater levels of the radiotracer in the bloodstream and help in greater brain uptake.

In the 5xFAD mice brain, there was a reasonable amount of uptake in regions that are known to contain Aβ plaques in these mice, as confirmed by our in vitro experiments (Figure 7). Regional brain uptake of [^18^F]flotaza was higher in the 5xFAD mice (Figure 7B) compared to the C57BL/6J mice (Figure 7C). The measured ratio of [^18^F]flotaza binding in the PET/CT images in different brain regions versus the cerebellum as a reference region was higher in the 5xFAD mice compared to C57BL/6J mice (Figure 7). The ratios were: TH/CB = 1.4 (5xFAD), 1.11 (C57BL/6J); HP/CB = 1.25 (5xFAD), 0.96 (C57BL/6J); FC/CB = 1.27 (5xFAD), 1.0 (C57BL/6J). The highest SUVR in the thalamus is consistent with in vitro results shown in Figure 6, although significantly lower in the in vivo PET experiment. Optimization of the in vivo binding in the 5xFAD mice will be carried out in future experiments.

### 2.7. Ex Vivo Autoradiography of Transgenic 5xFAD Mice

**Figure 8:**
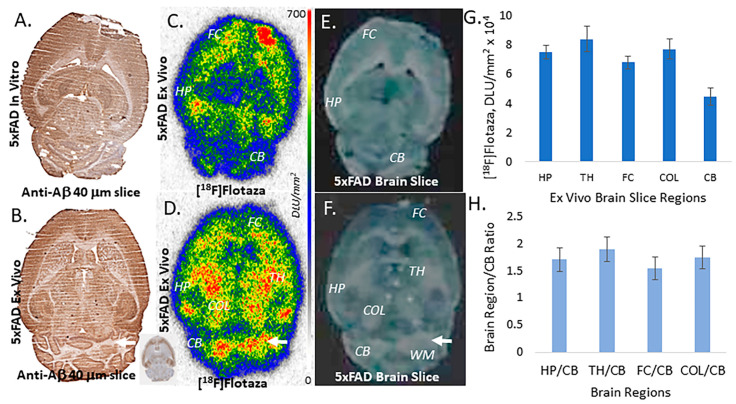
Ex vivo [^18^F]flotaza in transgenic 5xFAD mice: (**A**,**B**) Anti-Aβ immunostains of ex vivo 5xFAD mouse brain slices (40 μm thick); (**C**,**D**) Ex vivo [^18^F]flotaza autoradiographic images of 5xFAD mouse brain slices (40 μm thick) showing binding of [^18^F]flotaza in the hippocampus and midbrain regions (FC: frontal cortex; TH: thalamus; COL: colliculi; HP: hippocampus; CB: cerebellum). White arrow shows high nonspecific binding in cerebellar WM; (**E**,**F**) Scans of ex vivo 5xFAD mouse brain slice (40 μm thick) shown in (**C**,**D**). White arrow in (**F**) shows cerebellar WM; (**G**) Plot of ex vivo [^18^F]flotaza autoradiographic binding (DLU/mm^2^) in brain slice regions of the 5xFAD mice; (**H**) Brain regions to cerebellum ratio plot shows selective binding in TH (1.89), FC (1.54), HP (1.70), and COL (1.74).

After the PET study of the 5xFAD mice, the brains of select mice were excised and sectioned to provide horizontal brain slices. Because of the longer half-life of fluorine-18, sufficient radioactivity remains in the brain to provide adequate autoradiographs, as shown in Figure 8 and previously reported by us for other radiotracers. Brain slices were exposed on phosphor screens. After radioactive decay, select slices were immunostained for Aβ plaques (Figure 8A,B). The corresponding ex vivo binding of [^18^F]flotaza is seen in Figure 8C,D, and the scanned slices in Figure 8E,F.

The rank order of binding of [^18^F]flotaza in the different brain regions was similar to both in vitro and ex vivo experiments (Figure 6E and Figure 8G). The thalamus was still the highest binding region, followed by the hippocampus and frontal cortex. Significant binding in the colliculi became apparent in the ex vivo slices. There were regional variations within the cerebellum. Although it displayed the lowest amount of [^18^F]flotaza binding, the cerebellar WM (Figure 8D,F) had a significant amount of nonspecific binding. Brain regions to cerebellum ratios (Figure 8H, TH/CB = 1.89, FC/CB = 1.54, HP/CB = 1.70 and COL/CB = 1.74) in the ex vivo slices were significantly lower compared to the in vitro experiments (where alcohol was used in the buffer to wash away nonspecific binding) but was higher than the in vivo PET experiments. The ex vivo ratios were somewhat greater compared to the in vivo PET ratios for the various brain regions because of the greater accuracy of analysis of brain regions.

## 3. Discussion

We have developed [^18^F]flotaza as a high affinity and selective Aβ plaque imaging agent with an affinity of 1.65 nM for Aβ plaques measured in postmortem human AD brain slices [14]. Belonging to the “aza” class of compounds, it showed little binding to Tau in the postmortem human AD brain. Human postmortem studies in the cortex showed excellent [^18^F]flotaza binding in GM regions of the anterior cingulate that contain Aβ plaques. The same brain slices with WM in the corpus callosum exhibited very low binding of [^18^F]flotaza [14]. This binding of [^18^F]flotaza in the anterior cingulate was quantitatively correlated with adjacent brain sections that were immunostained, and the Aβ plaque positivity in each subject was calculated using QuPath procedures [17]. Very high ratios (>100) between the anterior cingulate and corpus callosum in the AD subjects were measured.

The hippocampus is the brain region where significant levels of Aβ plaques are found and may be associated with the initial formation and accumulation of the Aβ fibrils and the beginnings of neurodegeneration in AD [18,22]. Subregional hippocampal distribution of Aβ plaques in CA1, CA2, CA3, and subiculum have been examined in postmortem AD brains using immunostaining approaches [18,22]. Of these subregions, CA1 and subiculum were found to have higher levels of Aβ plaques, which is consistent with the high levels of Aβ in CA1. Using [^18^F]flotaza, a PET imaging radiotracer, we were able to confirm these findings of high levels of Aβ plaques in male and female AD subjects. There was a strong correlation between anti-Aβ immunostains and [^18^F]flotaza within subjects and across all subjects.

Although both female and male AD subjects exhibited high levels of [^18^F]flotaza in the hippocampus, there were subtle differences. In the GM, males exhibited slightly higher levels of [^18^F]flotaza binding compared to females, but these levels were not statistically significant (Figure 4A). A significant difference was observed in the WM between males and females (Figure 4B). All males consistently exhibited higher [^18^F]flotaza binding in the WM of the hippocampus compared to the females. White matter hyperintensities (WMH) have been reported in AD subjects associated with both Aβ plaques and tangles [27]. Correlations of WMH with Aβ and Tau show a progressive increase with Aβ as well as Tau, with a stronger correlation with Aβ and WMH [28]. Similar findings have been reported in Aβ+ cognitively normal elderly subjects. Demyelination of WM tracts has been reported to occur in AD, which compromises the integrity of WM and causes hypoxia, ischemia, and glutamate-induced excitotoxicity [29,30]. MRI studies have shown microstructural damage, structural disconnection, and topological abnormalities in AD [29,31]. The presence of Aβ in WM was reported to cause the additional deterioration and thinning of the cortical GM. These reports of lower [^18^F]flotaza binding in the WM of females may suggest lower Aβ-mediated binding due to greater demyelination of WM tracts compared to males WM. There are a number of reports on the greater vulnerabilities of female AD subjects [24]. Such evidence implicates sex differences playing a possible role in the greater deterioration of WM.

The majority of CN subjects, both males and females, did not exhibit any [^18^F]flotaza binding in GM and WM. Of the 32 CN subjects, one CN subject exhibited high binding of [^18^F]flotaza, which correlated with anti-Aβ immunostains: one subject with low levels and five subjects with higher than background levels. Increasing levels of [^18^F]flotaza (GM/WM) binding correlated with Braak staging [32] with each progressive stage until stage VI in females. Thus, imaging using [^18^F]flotaza seems appropriate for Aβ staging in the AD postmortem brains. The AD subjects spanned two decades (for males) and three decades (for females). In this limited age range, there was a poor correlation of age with the [^18^F]flotaza GM/WM ratios both for males (R^2^ = 0.0543; Spearman’s correlation ρ = 0.1821, *p*-value = 0.5489; Figure 4C) and for females (R^2^ = 0.1698; Spearman’s correlation ρ = −0.2238, *p*-value = 0.4196; Figure 4D). Similar low correlations have been previously reported in a larger cohort of postmortem hippocampal studies of AD subjects [23].

In order to further the in vivo use of [^18^F]flotaza, the radiotracer was also evaluated in transgenic 5xFAD mice brains expressing Aβ plaques that are a useful model of human AD [19,20,21]. In vitro brain studies, PET/CT in vivo imaging, and ex vivo autoradiographic analyses of brain slices were carried out in the 5xFAD mice in order to evaluate the suitability of [^18^F]flotaza for in vivo PET imaging of Aβ plaques. Abundant Aβ plaques in the 5xFAD mice have now been well-studied using immunostaining [20], fluorescent [21], and PET imaging probes [15,26]. Our results here with [^18^F]flotaza show very high in vitro binding to Aβ plaques in the various brain regions of the 5xFAD mice. Using the cerebellum as the reference region, brain slice studies reveal very high ratios of binding to the thalamus, hippocampus, lateral septal nuclei, and frontal cortex and is consistent with our observations in the human AD anterior cingulate [14] and hippocampus as previously mentioned. [^18^F]Flotaza binding is strongly correlated with anti-Aβ IHC in the mice. Similar to our previous results with [^124^I]IBETA, the thalamus showed the highest levels of [^18^F]flotaza binding [15]. Compared to hippocampal Aβ immunostains, thalamic Aβ plaques appear diffuse (Figure 6C,D) and may be structurally different from the hippocampal Aβ plaques that are formed early in AD [33,34]. The selectivity of [^18^F]flotaza to Aβ plaques in the thalamus is evident by the complete absence of any binding in the WT C57BL/6J mice brain slices (Figure 6F,G). Thus, thalamic Aβ plaques are abundant in the 5xFAD mouse model and have not been identified by previous techniques [35]. This is not off-target binding since thalamic binding was also previously reported using [^124/125^I]IBETA [15]. [^18^F]Florbetaben, a related PET radiotracer similar to [^18^F]flotaza, shows some thalamic binding in an APPswe/PS2 transgenic mice PET study [36]. Due to the higher iron content associated with Aβ plaques in the thalamus, in vivo magnetic resonance imaging (MRI) was used to study transgenic APP/PS1 mice [37]. Autopsy studies in the human AD brain have found Aβ plaques in the thalamus, with higher amounts in the anteroventral nucleus [38]. Thalamic involvement in the human phases of Aβ deposition has been shown [39], although human PET imaging of radiotracers has been less pronounced [40,41]. Thioflavin staining and immunohistochemistry have been less efficient in identifying the high levels of thalamic Aβ plaques in transgenic mice models of AD [42,43]. Detailed nanoscale structure of thalamic versus hippocampal Aβ plaques in the 5xFAD model may shed additional light on differences in radioligand binding [44]. In this preliminary study, female 5xFAD mice showed higher levels of Aβ plaques compared to males (Figure 6E).

[^18^F]Flotaza was able to cross the blood–brain barrier in the 5xFAD mice. Similar to lipophilic molecules, the excretion of [^18^F]flotaza occurred via the liver and eventually to the urinary bladder (Figure 7A). The low activity in the brain was retained in the 5xFAD mice (Figure 7B,C) compared to the C57BL/6J (Figure 7D), suggesting Aβ plaque-mediated retention of [^18^F]flotaza. The cerebellum had the least amount of [^18^F]flotaza, as expected, while regions in the cerebrum, such as the frontal cortex, thalamus, and hippocampus, were clearly delineated in the PET scans. [^18^F]Flotaza appears to show improved regional brain binding in this mouse model compared to the reported fluoropegylated radiotracers such as [^18^F]florbetaben [21,26]. In vivo microPET imaging of the Tg2576 mouse model using the radiotracer [^11^C]PIB, which binds to amyloid plaques, has been reported [45]. The ratio of the frontal cortex to the cerebellum was not found to be significantly different compared to wild-type mice. Similar findings of low binding to transgenic mice by [^11^C]PIB have also been recently reported [46]. A high-resolution microPET study with ^18^F-FDDNP in transgenic rats revealed selective frontal cortex to cerebellum binding compared to wild type [47]. Although in vitro autoradiographic studies on transgenic mice demonstrate amyloid plaque accumulation [48], successful in vivo imaging has been difficult. More recent efforts have been more promising [49,50,51]. However, reported methods still suffer from poor SUV values in the AD mice, and binding appears to be strain-selective.

Ex vivo brain studies of the 5xFAD mice after the PET scan further confirmed the regional distribution of [^18^F]flotaza in Aβ-rich regions (Figure 8). The relative binding profile in the ex vivo brain slices was similar to the in vivo PET scan. It should be noted that cerebellar WM had a significant amount of nonspecific binding. Therefore, lateral cerebellar lobes consisting more of the GM should be used as a reference region in the PET scans of [^18^F]flotaza. Typical SUVR reported for the various Aβ imaging agents in AD patients range from 1.2 to 1.5. Our results with [^18^F]flotaza fall in this range for the 5xFAD mice. Greater clearance of [^18^F]flotaza from nonspecific WM regions in humans may be expected with the potential of yielding higher SUVRs.

Radiation dosimetry of [^18^F]flotaza in WT and 5xFAD mice using PET/CT is planned in order to assess suitability for translation to humans using our previously reported methods [52]. Based on previously reported data, we expect the kidney and urinary bladder to be the critical organs. Subsequent steps will include toxicity evaluation in order to obtain an Investigational New Drug (IND) approval from the Food and Drug Administration (FDA) to advance to the next phase of the project, i.e., a safety and efficacy study in humans.

Within this study, the limitation exists of a small number of AD subjects analyzed. Additionally, there may be small inter-subject variations in the brain tissue, which may have caused variations in the GM binding of [^18^F]flotaza. The variability observed in [^18^F]flotaza binding to WM in both male and female AD subjects will require additional subjects. Whether this variation is only seen in the chosen subjects or if it is a common trend in all AD subjects is difficult to determine with the size of the samples. Additional 5xFAD mice in vivo studies with intravenous administration of [^18^F]flotaza are needed to assess the SUVR more fully. However, it is important to note that although the 5xFAD mice model is a suitable representation of Aβ plaques and PET evaluation, these findings may not be accurately and reliably translated to PET studies of Aβ plaques in human AD.

## 4. Materials and Methods

### 4.1. General Methods

Fluorine-18 was purchased from PETNET, Inc. (Makati, Metro Manila) Fluorine-18 labeled [^18^F]flotaza was prepared as reported previously [14]. Capintec CRC-15R dose calibrator and Capintec Caprac-R well-counter were used for radioactivity measurements. Thin layer chromatography of radioligands was scanned on an AR-2000 imaging scanner (Eckart & Ziegler, Berlin, Germany). Cyclone phosphor autoradiographic imaging system (Packard Instruments Co., Meriden, CT, USA) and Optiquant Imaging System software, version 5.0 were used for analysis. Specialty chemicals PIB was purchased from ABX Inc., Radeberg, Germany.

### 4.2. Postmortem Human Brain

Human postmortem brain tissue sections of HP (CA1 plus subiculum), 10 mm thick on Fisher slides, were obtained from Banner Sun Health Research Institute (BHRI), Sun City, AZ, brain tissue repository for in vitro experiments [25]. Brain tissue samples from AD and cognitively normal (CN) subjects were selected based on their cognitive status during life and the presence or absence of end-stage pathology. Age and gender-matched AD brain and CN brain tissue samples were used for the study. A total of CN, n = 32; 16 males (age 71–97) and 16 females (age 53–95) and AD, n = 28; 13 males (age 70–91) and 15 females (age 59–93) were used in this study (Table 1). Brain sections were stored at −80 °C. All human studies were approved by the respective Institutional Review Boards. All postmortem human brain studies were approved by the Institutional Biosafety Committee of the University of California, Irvine.

### 4.3. Human Aβ Plaque Imaging Autoradiography

Purified [^18^F]flotaza was used for autoradiographic studies [14]. Human brain sections (10 μm thick) were placed in a glass chamber and preincubated in a PBS buffer for 10–15 min. The brain sections were placed in glass chambers and incubated with [^18^F]flotaza (approximately 74–111 kBq/mL; 0.5–0.75 nM; specific activity > 35 GBq/μmol)) in 40% ethanol–PBS buffer at 25 °C for 1.5 h. The slices were then washed with cold PBS buffer (1 × 5 min), 60% ethanol–PBS buffer (2 × 5 min), PBS buffer (1 × 5 min), and cold deionized water (2 min), respectively. Nonspecific binding was measured in the presence of 10 μM PIB. The brain sections were air-dried and exposed overnight on a phosphor film (Multisensitive Medium MS, PerkinElmer, Waltham, MA, USA). The apposed phosphor screens were read and analyzed by OptiQuant acquisition and the Cyclone Storage Phosphor System (Packard Instruments Co., Boston, MA, USA). The regions of interest (ROIs) were drawn on the slices, and the extent of binding of [^18^F]flotaza was measured in DLU/mm^2^ using the OptiQuant acquisition and analysis program.

### 4.4. Immunohistochemistry

Immunostaining of all brain sections was carried out by the University of California-Irvine Pathology services using Ventana BenchMark Ultra protocols. For Aβ plaques, slices from all subjects were immunostained with anti-Aβ Biolegend 803015 (Biolegend, San Diego, CA, USA), which is reactive to amino acid residue 1–16 of β-amyloid. All IHC-stained slides were scanned using the Ventana Roche instrumentation and analyzed using QuPath [17,53,54]. QuPath (version QuPath-0.4.2) was used for quantitative analysis of scanned brain slices.

Using QuPath, a pixel threshold was created to outline the IHC images. Several annotations were made for Aβ plaques in the grey matter regions of the IHC brain slices of each subject. Approximately 20–25 annotations were made for Aβ plaques by means of visual identification of being an Aβ plaque. Negative annotations (approx. 15) with no Aβ plaques were drawn in each subject. The pixel classifier was run (using either random trees or artificial neural networks), and the entire brain slice of each subject was generated by the pixel classifier and saved as a new image. To measure the area of Aβ plaques for each new pixel-classified brain slice, regions of interest (ROI) for grey matter and white matter were drawn on the new classified image. This ROI was then run through the classifier again and asked to measure all annotations. The area of Aβ plaques in the ROI, total area, and percent of Aβ were obtained for each brain slice [17]. This percent positivity of Aβ plaques was used to correlate with autoradiography measures.

### 4.5. Animals

All animal studies were approved by the Institutional Animal Health Care and Use Committee of the University of California-Irvine. The C57BL/6 adult male and female mice used in this study (28–40 g; 12-month-old) were purchased from Jackson Laboratory and housed under controlled temperatures of 22 °C ± 1 °C in a 12-h light–dark cycle starting at 6:00 a.m. with water and food chow ad libitum. The 5xFAD transgenic line of mice (MMRRC hemizygous 4 male and 4 female; 12-month-old) were purchased from Jackson Laboratory (female mice weighed 20–28 g and male mice weighed 26–38 g). All mice were housed in standard cages.

### 4.6. 5xFAD Transgenic Mice In Vitro

All experiments were carried out in accordance with the Institutional Animal Care and Use Committee at the University of California, Irvine, and were consistent with Federal guidelines. Male and female hemizygous 5xFAD mice obtained from MMRRC JAX were used for in vitro and in vivo studies. Horizontal brain slices were sectioned (10 μm thickness) on a Leica 1850 Cryostat and collected on Fisher slides. The slides contained three to four brain sections placed in separate glass chambers (six slides per chamber) and were preincubated in a PBS buffer for 15 min. The preincubation buffer was discarded. The brain slices were treated with [^18^F]flotaza in 40% ethanol–PBS buffer pH 7.4 (60 mL, 5 kBq/mL). The chambers were incubated at 25 °C for 1.25 h. Nonspecific binding of [^18^F]flotaza was measured in separate chambers using IBETA (10 µM). The slices were then washed with cold PBS buffer, 90% ethanolic PBS buffer twice, PBS buffer, and cold water. The brain sections were air-dried and exposed overnight on a phosphor film. The regions of interest (ROIs) were drawn on the slices, and the extent of binding of [^18^F]flotaza was measured in DLU/mm^2^.

### 4.7. PET/CT Studies In Vivo

Animals had free access to food and water during housing. All animals were fasted for 18–24 h prior to PET imaging. In preparation for the scans, the mice were induced into anesthesia with 3% isoflurane. Inveon preclinical Dedicated PET (Siemen’s Inc., Munich, Germany) was used for the MicroPET studies, which had a resolution of 1.45 mm [55]. The Inveon PET and MM CT scanners were placed in the “docked mode” for combined PET/CT experiments (Siemens Medical Solutions, Knoxville, TN, USA). A Sigma Delta anesthetic vaporizer (DRE, Louisville, KY, USA) was used to induce and maintain anesthesia during injections and PET/CT acquisitions. [^18^F]Flotaza was taken in 10% alcoholic sterile saline (0.9% NaCl injection, United States Pharmacopeia) for injections into mouse models for PET/CT studies. [^18^F]Flotaza was injected retro-orbitally (0.9 MBq) under 2% isoflurane anesthesia. The mice underwent 30-min PET scans (90 min postinjection) in a supine position accompanied by a 7-min CT scan, which was used for reconstruction and attenuation correction of PET images. The CT images were reconstructed with a cone beam algorithm (bilinear interpolation, Shepp–Logan filter) into 480 × 480 × 632 image arrays with a 206 μm pixel size. Following the reconstruction, the CT images were spatially transformed to match the PET images. In addition to being reconstructed into an image, these CT data were used for attenuation correction of PET images. PET imaging data were quantified as standard uptake values (SUVs), and using values of the nonspecific binding region, cerebellum, ratios (SUV ratios, SUVRs) of [^18^F]flotaza were calculated using our reported procedures for [^124^I]IBETA [15].

### 4.8. Ex Vivo Autoradiography

After [^18^F]flotaza in vivo PET/CT experiments in control and 5xFAD mice, select mice were killed, and the brain was excised. The brains containing [^18^F]flotaza were rapidly frozen at −80 °C for approximately 10 min, followed by 10 min in the Leica cryotome at −20 °C. Similar procedures were previously reported for other radiotracers involved in correlative studies between in vivo and ex vivo measurements of brain uptake in small animal models [56]. Horizontal brain sections (10 µm to 40 μm thick) containing the thalamus, subiculum, cortex, striatum, hippocampus, and cerebellum were cut using the Leica cryotome. The sections were rapidly air-dried and subsequently exposed to phosphor films overnight. Adjacent sections were immunostained with Aβ-antibodies. The regions of interest (ROIs) were drawn on the slices, and the extent of binding of [^18^F]flotaza was measured in DLU/mm^2^.

### 4.9. Image Analysis

All regions of interest (ROIs) in the GM and WM autoradiographic images of [^18^F]flotaza were quantified using measures (DLU/mm^2^). The immunostained sections were analyzed using QuPath. GM and WM binding of [^18^F]flotaza in AD and CN subjects were measured, and GM/WM ratios of AD and CN were compared. Using the ratio method would be akin to in vivo PET methods, where the SUV between the target region is compared to a nonspecific binding reference region as a ratio and expressed as SUVR [15,19].

All in vivo images were analyzed using Inveon Research Workplace (IRW) software version 2.1, (Siemens Medical Solutions, Knoxville, TN, USA) and PMOD Software version 3.0 (PMOD Technologies, Zurich, Switzerland). Whole-body PET/CT images were analyzed using the IRW software for [^18^F]flotaza uptake and any other CT anomalies in the whole-body images. For additional brain quantitative analysis, brain images were also analyzed using PMOD, with PET images co-registered to a mouse brain MRI template [19]. The magnitude of [^18^F]flotaza in each volume of interest, VOI (in kBq/mL), was measured. The cerebellum was used as a reference region to calculate the ratio of target brain regions to reference regions.

### 4.10. Statistical Analysis

Group differences between AD and CN subjects were assessed using average GM/WM ratios and were determined using Microsoft Excel 16 and GraphPad Prism 9. Statistical power was determined with Student’s *t*-test, and *p*-values of <0.05 were considered to indicate statistical significance. Spearman’s correlation was carried out to assess the effects of aging.

## 5. Conclusions

In summary, [^18^F]flotaza is a new PET radiotracer for imaging Aβ plaques in the human brain. The findings reported here indicate that [^18^F]flotaza may be expected to give a significantly higher target to nontarget ratios in PET studies. If confirmed, the higher binding of [^18^F]flotaza to Aβ plaques may allow the detection of lower levels of Aβ plaques at earlier times more precisely, thus enabling etiology studies, earlier diagnosis, and treatment evaluation [57,58]. Despite previous unsuccessful efforts [59], reduction in the accumulation of Aβ amyloid plaques continues to be investigated as a therapeutic approach for AD [10,60,61]. Recent efforts with Lecanemab to reduce Aβ plaque load in early AD patients appear promising [9,10]. Radiolabeled [^125^I]IPC-Lecanemab has been reported to bind to Aβ aggregates, and in vivo, imaging of the [^125^I]IPC-Lecanemab along with small molecule [^18^F]flotaza may be useful in treatment strategies of AD patients [62].

## Figures and Tables

**Table 1:** Patient Samples and Data *.

Subjects, N	CERAD Pathology	Gender	Age Range, Mean ± SD	PMI, Hours	Brain Region ^1^	Plaque Total	Tangle Total	LB	Braak Score
16	CN	Male	71–97 (79.9 ± 8.55)	2–5.4	HP	0–5.5	0–6	0	I–III
16	CN	Female	53–95 (80.4 ± 13.1)	2.1–4.8	HP	0–10	0.5–6.5	0	I–III
13	AD	Male	70–91 (80.4 ± 5.98)	2.3–4.8	HP	14–15	10–15	0	V–VI
15	AD	Female	59–93 (81.3 ± 9.26)	1.8–5	HP	10–15	12–15	0	V–VI

* Frozen brain samples were obtained from Banner Sun Health Institute, Sun City, Arizona [24]; CN = cognitively normal and may include mild cognitive impairment (MCI) subjects; AD = Alzheimer’s disease; PMI: Postmortem interval in hours. Plaque total: Includes neuritic, cored, and diffuse in the frontal, temporal, parietal, hippocampal, and entorhinal cortex. Semi-quantitative scores of none, sparse, moderate, and frequent were converted to numerical values 0–3 for each region and summed to provide Plaque total; Tangle total: neurofibrillary tangle density in frontal, temporal, and parietal lobes, hippocampal CA1 region, and entorhinal cortical regions. Numerical values 0–3 for each region were summed to provide the Tangle total; Braak score: Braak neurofibrillary stage (0–VI) defined in [25]. ^1^ HP: hippocampus containing subiculum; Brain slices (10 μm thickness) were obtained from the chunks of frozen tissue on a Leica 1850 cryotome, collected on Fisher slides and stored at −80 °C.

## Data Availability

The data that supports the findings of this study are available from the corresponding author for discussions upon reasonable request.
